# Prenatal Diagnosis and Fetomaternal Outcomes of Two Cases with Placental Chorioangioma

**DOI:** 10.1155/2013/926743

**Published:** 2013-10-22

**Authors:** Burcu Artunc Ulkumen, Halil Gursoy Pala, Nalan Nese, Yesim Baytur

**Affiliations:** ^1^Obstetrics and Gynecology Department, School of Medicine, Celal Bayar University, 45030 Manisa, Turkey; ^2^Pathology Department, School of Medicine, Celal Bayar University, 45030 Manisa, Turkey

## Abstract

Placental chorioangiomas greater than 4 cm in diameter are rare placental tumors. They have adverse fetomaternal outcomes. We present our experience with two cases having a giant angioma and review the relevant literature.

## 1. Introduction

Chorioangiomas—also known as placental hemangiomas—are benign hamartomatous growth of the placenta comprising vascular tissue. The classification is controversial due to its pathologic features and clinical implications [1]. Some authors call placental hemangiomas as true nontrophoblastic neoplastic tissues [2]. As this abnormal vascular growth seems like the native placental tissue and it does not metastasize, some authors call it just as placental hamartoma [1]. The general incidence is approximately 0.6% [2]. However, giant chorioangiomas with a diameter of greater than 5 cm are very rare with an incidence of 1/16.000–1/50.000 pregnancies [1]. Small tumors do not cause any adverse outcomes and they are asymptomatic. In contrast, the tumors greater than 5 cm may lead to several complications such as preterm labor, placental abruption, polyhydramnios, fetal hydrops, intrauterine growth restriction, cardiac failure, and even fetal death. Perinatal mortality is as high as 30–40% [1]. Therefore, prenatal diagnosis is important to follow up these pregnancies closely. We report here two cases with giant placental chorioangiomas and review the relevant literature.

**Case 1**

**Figure 1:**
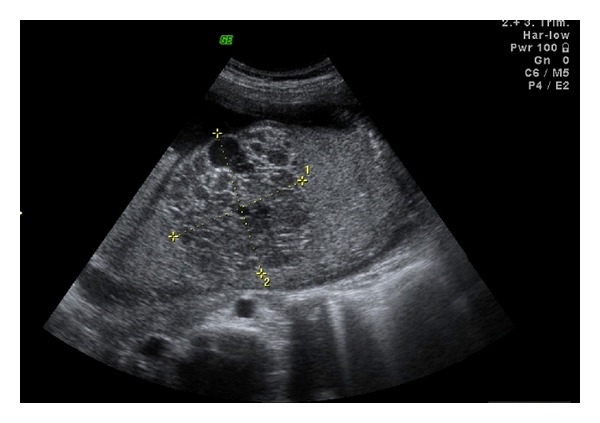
The gray-scale ultrasonography of Case 1 showing 7 × 7 × 6 cm hypoechoic, well-circumscribed intraplacental mass with some anechoic areas.

**Figure 2:**
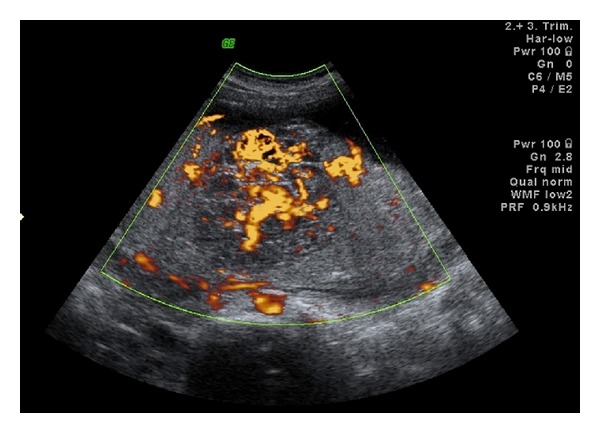
The power Doppler ultrasonography of the placental angioma of Case 1 showing rich vascular supply within.

**Figure 3:**
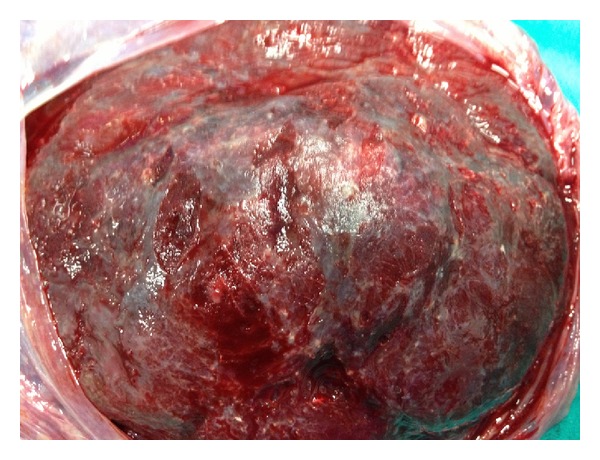
The image of the placenta from Case 1. The mass is protruding from its surface with rich vascular network.

**Figure 4:**
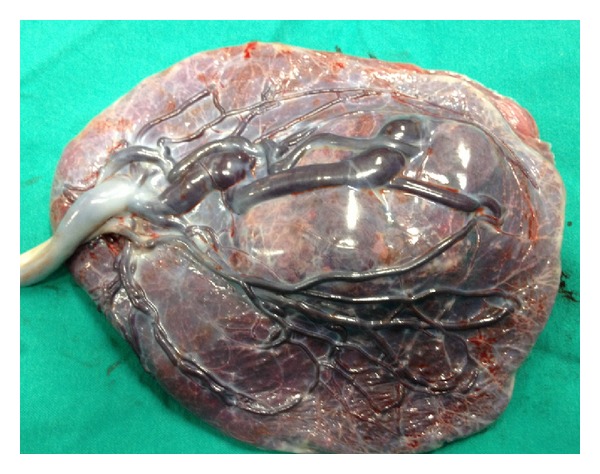
The photograph of the maternal surface of the placenta. The typical localization of the tumor is seen near the umbilical cord insertion.

A 32-year-old primigravid woman was admitted to our perinatology department due to a placental mass. She had an uneventful prenatal course until the diagnosis of a placental mass at 36 gestational weeks. She did not have any complaints. Her vital signs were within normal range. The biometric calculation of the baby was 34 gestational weeks. Bilateral uterine arteries and umbilical artery blood flow were normal. Amniotic fluid was in normal amount. The obstetric sonography image showed a 7 × 7 × 6 cm hypoechoic, circumscribed intraplacental mass with some anechoic areas (Figure 1). Power Doppler examination showed dense blood flow within the mass (Figure 2). At 38 gestational weeks, the patient had cesarean delivery due to oblique presentation of the baby. The female newborn was 2825 gr with 8/10 Apgar scores at 1st and 5th minutes, respectively. The placenta was 146 g and 20 × 16 × 16 cm in size. The three-vessel umbilical cord with 12 cm in length and 0.8 cm in diameter was eccentrically placed. A single 7 × 6 × 5 cm spherical solid mass was extending from the maternal surface to the fetal surface (Figures 3 and 4). The mass had dense vessels. Microscopic examination revealed tumoral mass containing small- and medium-sized vessels with prominent endothelial tissues and intervillous fibrin deposition. The histologic evaluation was consistent with chorioangioma.

**Case 2**

**Figure 5:**
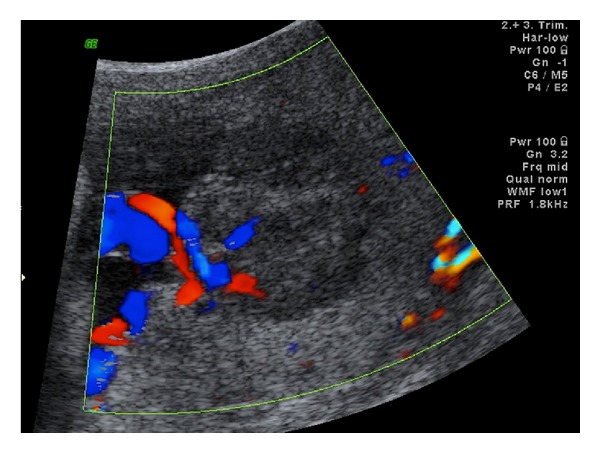
The color Doppler ultrasonography of Case 2 showing the typical place of the tumor next to the umbilical cord insertion.

A 37-year-old primiparous woman with a prior cesarean history was admitted to our perinatology department due to a placental mass. She had an uneventful prenatal course until the diagnosis of a placental mass at 26 gestational weeks. At the time of diagnosis, she had no complaints. Her vital signs were within normal range. The biometric calculation of the baby was 26 gestational weeks. Bilateral uterine arteries and umbilical artery blood flow were normal. The obstetric sonography image showed a 5 × 4 × 4 cm hypo-hyperechoic, well-circumscribed intraplacental mass with some anechoic areas in it. Power Doppler examination showed the typical localization of the mass near to the umbilical cord insertion with rich blood flow within the mass (Figure 5). Until 38th gestational week, she was asymptomatic. Any pregnancy complication was noticed. The pregnancy was monitored every 2-3 weeks. At 38 gestational weeks, the patient had cesarean delivery. The male newborn was 3200 gr with 8/10 Apgar scores at 1st and 5th minutes, respectively. The placenta was 690 g and 18 × 17 × 14 cm in size. The three-vessel umbilical cord with 8 cm in length and 1 cm in diameter was eccentrically placed. A single 5.5 × 5 × 4 cm spherical solid mass was extending from the maternal surface to the fetal surface. The mass was rich in blood vessels. Microscopic examination revealed tumoral mass containing congested and dilated numerous blood vessels with prominent endothelial tissues and intervillous fibrin deposition. There was wide ischemic necrosis. The histologic evaluation was consistent with chorioangioma.

## 2. Discussion

Chorioangiomas are the most common benign nontrophoblastic tumors of the placenta and originate from the chorionic tissue. Tumors smaller than 4-5 cm in diameter are usually asymptomatic and do not cause any adverse perinatal outcomes. However, tumors greater than 5 cm are associated with some perinatal complications. The mechanism is probably because that the big tumors act like arteriovenous shunts by leading to fetal congestive heart failure, hydrops, and even in utero death [1–3]. Wehrens et al. proposed that this arteriovenous shunting initiates a chain reaction in the hemodynamic system of the fetus for the compensation of these changes [4]. Sudden in utero death, fetal distress, preterm birth, polyhydramnios, and elevated serum alpha-fetoprotein (AFP) levels are the main perinatal complications. Elevated maternal serum AFP levels during the second trimester screening may also alarm about the possibility of the placental angioma. In utero growth restriction (IUGR) is the other expected complication, because the proper blood supply can not be achieved by the fetus. In a series with 6 chorioangioma cases, there was one pregnancy with IUGR [5]. And also Zanardini et al. found that 6 patients had IUGR within their series with 19 cases [6]. However, some pregnancies with giant angiomas may end up in uneventful prenatal outcomes as in our cases.

Small angiomas undetected during prenatal sonography can be only established by careful pathologic examination. Guschmann et al. showed in a retrospective analysis of 136 angiomas that only less than half were detected during prenatal period. Moreover, they proposed that the angioma incidence rises with maternal age and is found more often in twin pregnancies. Preterm labor is more frequent in angiomas. There are more female babies with angiomas in their placenta [7]. 

Doppler sonography is the main tool in differential diagnosis between angioma and placental hematoma, teratoma, and even myoma uteri. Great blood flow in the placental mass and in the lacunar anechoic areas of the mass or a gross feeding vessel within the placental mass favors the diagnosis of chorioangioma [1–3]. Similarly in our cases, there was abundant blood flow in the lacunae of the placental tumor (Figure 2). Placental teratomas are cystic or solid masses with calcifications on sonographic examination. Teratomas do not have rich vascular supply. In contrast to angiomas, echogenicity of hematomas differs from time to time as the coagulation process occurs. 

Histologically, three different forms may be seen in chorioangiomas: angiomatous, cellular, and degenerative forms [1]. The angiomatous form is the most common. The tumor of our cases had angiomatous structure with small- and medium-sized vessels and numerous endothelial tissues.

Fetal treatment of the chorioangioma is controversial. Small tumors can be managed expectantly. However, for some complications, treatment can be thought of. Amniodrainage of the excessive amniotic fluid is an option in case of polyhydramnios. In uterine transfusion and laser coagulation of the vascular shunts are the other management options [3, 4, 6].

The key point in the follow-up of these patients is to keep in mind that at anytime the fetus can get distress, and at anytime fetal hydrops and fetal congestive heart failure can occur. The proper timing of the labor is controversial and primarily depends on the fetomaternal complications. Unless these complications occur, the patients should be monitored closely, at least every month for small tumors and every 1-2 weeks for the greater ones.
